# Association between Duration of Exercise (MET Hours per Week) and the Risk of Decreased eGFR: A Cross-Sectional Study Based on a Large Chinese Population

**DOI:** 10.1155/2019/5874603

**Published:** 2019-03-21

**Authors:** Jie Wang, Yijun Li, Kang Chen, Wenhua Yan, Anping Wang, Weiqing Wang, Zhengnan Gao, Xulei Tang, Li Yan, Qin Wan, Zuojie Luo, Guijun Qin, Lulu Chen, Yiming Mu

**Affiliations:** ^1^Department of Endocrinology, Chinese PLA General Hospital, Beijing, China; ^2^Medicine School of Nankai University, China; ^3^Shanghai National Research Centre for Endocrine and Metabolic Diseases, State Key Laboratory of Medical Genomics, Shanghai Institute for Endocrine and Metabolic Diseases, Ruijin Hospital, Shanghai Jiaotong University School of Medicine, Shanghai, China; ^4^Dalian Central Hospital, Dalian, Liaoning, China; ^5^First Hospital of Lanzhou University, Lanzhou, Gansu, China; ^6^Zhongshan University Sun Yat-sen Memorial Hospital, Guangzhou, Guangdong, China; ^7^Southwest Medical University Affiliated Hospital, Luzhou, Sichuan, China; ^8^First Affiliated Hospital of Guangxi Medical University, Nanning, Guangxi, China; ^9^First Affiliated Hospital of Zhengzhou University, Zhenzhou, Henan, China; ^10^Wuhan Union Hospital, Huazhong University of Science and Technology, Wuhan, Hubei, China

## Abstract

**Background:**

Physical activity is effective in preventing chronic diseases. However, the impact of different durations of exercise on human health is unknown, especially among people with diabetes or prediabetes.

**Objective:**

To explore the relationship between high MET hours per week and the change in glomerular filtration rate (eGFR) in the total population and different subgroups.

**Methods:**

A total of 43767 individuals from eight provinces, in China, were recruited. Logistic analysis was used to investigate the association. Participants were divided into 3 groups based on MET hours per week. The primary outcome was an eGFR ≤ 90 mL/min/1.73 m^2^.

**Results:**

The average eGFR was 100.10 (92.43-106.43) mL/min/1.73^2^. Logistic regression analysis revealed that more than 7.5 MET hours per week (equivalent to more than 150 minutes of moderate-intensity of exercise) was associated with the higher risk of the decreased eGFR even after adjusting for confounding factors (7.5 to <21: OR = 1.18, 95% CI [1.09, 1.29]; ≥21: OR = 1.12, 95% CI [1.05, 1.19], *p* for trend: 0.0047). After adjusting for confounding factors, in stratified analyses, there still existed a significant relationship among participants aged from 55 to less than 65 years, but not among participants younger than 55 or older than 65 years. Similarly, there existed a positive association between high MET hours per week and the decreased eGFR in participants without diabetes and prediabetes, but not in participants with diabetes or prediabetes, and the interactions of age and diabetic states were found. However, there was no significant difference in women or men.

**Conclusions:**

More than 7.5 MET hours per week (equivalent to more than 150 minutes per week or 60 minutes per day of moderate-intensity exercise) was associated with decreased eGFR among participants aged from 55 to less than 65 years and participants without diabetes and prediabetes, but not among participants aged younger than 55 years and older than 65 years and participants with diabetes or prediabetes. The importance of planning individualized physical activities is highlighted.

## 1. Introduction

Haskell et al. confirmed that moderate physical exercise can help with weight loss and the reduction of the risk of cardiovascular diseases (CVD) [1]. Physical exercise is now viewed as one of the treatment options for diabetes mellitus (DM) and CVD. The World Health Organization (WHO) recommends that individuals perform at least 150 minutes of moderate-intensity exercise or 75 minutes of high-intensity exercise, weekly, to maintain physical function and health [2]. However, there are still not enough studies involved in the relationship between physical activity and chronic disease based on a Chinese population, especially people in different health conditions.

Kidney disease, as one of the main causes of death, can lead to an increased incidence of CVD [3]. It is reported that in developing countries, chronic kidney disease is associated with high morbidity. The estimated number of people with chronic kidney disease, in China, has reached 119.5 million in 2011 [4, 5]. However, the majority of people with renal dysfunction are unaware of it and still do more exercise to keep fit. Hence, it is significant to identify the risk factors for renal dysfunction, for prevention as well as early treatment.

There are some studies that showed moderate-intensity exercise could be beneficial to reduce the risk of CVD in patients with chronic kidney disease [6]. However, O'Keefe et al. found that participants in long-term high-intensity physical activity increased the risk of atrial fibrosis and atherosclerosis [7]. Additionally, Hiraki et al. found that moderate-intensity exercise did not improve the renal function of patients with chronic kidney disease [8]. A decreased estimated glomerular filtration rate (eGFR) and increased urinary protein-to-creatinine ratio were observed after high-intensity exercise in athletes and healthy individuals [9, 10]. But there is still a lack of evidence on the relationship between metabolic equivalent of energy (MET) (MET hours per week), a measure of physical activity, and eGFR. Therefore, the aim of the cross-sectional study is to investigate the relationships between MET hours per week and eGFR as well as the relationships in different subgroups, based on a large Chinese population.

## 2. Materials and Methods

### 2.1. Participants

**Figure 1:**
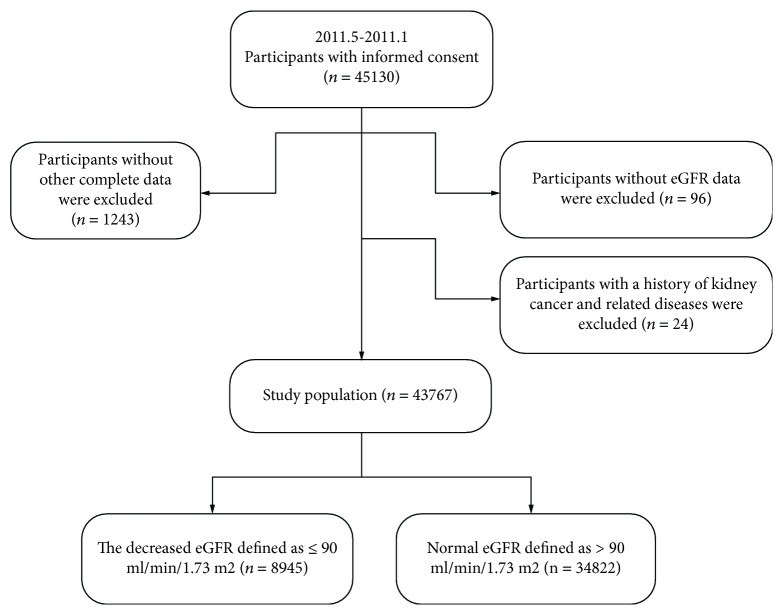
Selection of the study population.

This cross-sectional study was part of the REACTION Study, an ongoing longitudinal study, designed to investigate the association between diabetes and the risk of cancer among the Chinese population, reported previously [11]. Participants aged 40 years and older were recruited between May 2011 and December 2011. This study used a cluster random sampling method and was conducted in the Gansu, Guangxi, Guangdong, Henan, Hubei, Liaoning, Shanghai, and Sichuan provinces, in China. Initially, a total of 45,130 participants were recruited. Participants with missing information and a history of kidney cancer and related diseases and without eGFR data were excluded as shown in Figure 1. Finally, a total number of 43767 individuals were included in the cross-sectional study.

### 2.2. Data Collection

Data were collected by the same trained staff, according to standardized operational procedures. All the participants completed a standard questionnaire with the assistance of the trained staff. The questionnaire included education level, lifestyle, physical activity, smoking status, drinking status, medical history, and family history of tumors and DM. Regular smokers were defined as those who smoked at least one cigarette per day. Occasional smokers were participants who smoked less than one cigarette per day or less than 7 cigarettes per week. Regular drinkers were defined as participants who had consumed alcohol at least once a week for over six months. Occasional drinkers were defined as participants who drank less than once a week. Height and weight were clinically measured (when participants were wearing light clothing), and body mass index (BMI) was calculated using the formula BMI = weight (kg)/height (m)^2^. On the standard questionnaire, all participants provided their average amounts of time per week that they carried out the different groups of activities, including low-intensity, moderate-intensity, and vigorous-intensity activities based on the short version of the International Physical Activity Questionnaire (IPAQ). According to IPAQ, the energy expended for different intensity exercises was estimated in MET hours per week, and all these different intensity exercises were also summed to account for the total energy expenditure weekly.

After a resting period of five minutes, participants' blood pressure and pulse were measured three times with intervals of one minute in a seated position. The pulse rate was measured while the blood pressure was recorded. After at least 12 hours of fasting overnight, the first fasting blood samples of all the participants were obtained. Patients without a history of DM underwent a 75 g oral glucose tolerance test; they were required to drink 300 mL of a glucose solution containing 75 g of glucose within 5 minutes, and after 2 hours, the second venous blood sampling was performed. All blood samples were centrifuged for 30 minutes and stored at -80°C by the professional staff. Blood glucose, blood lipids, triglycerides (TGs), high-density lipoprotein (HDL), low-density lipoprotein (LDL), and serum creatinine were detected by the automatic enzyme method. This cross-sectional study is part of the REACTION study, using the baseline data, and the design and methodology of the REACTION study have previously been described in detail [11, 12].

### 2.3. Definition of Variables

The variables were defined as follows: hypertension (any self-reported history of hypertension or systolic blood pressure (SBP) ≥ 140 mmHg or diastolic blood pressure (DBP) ≥ 90 mmHg), diabetes (fasting blood glucose (FBG) ≥ 7.0 mmol/L and postprandial blood glucose (PBG) ≥ 11.1 mmol/L simultaneously or any self-reported history of diabetes), cardiovascular events (any self-reported history of coronary heart disease, stroke, and myocardial infarction), any self-reported family history of tumors, and any self-reported family history of DM. According to the WHO criteria, prediabetes was defined as follows: 6.1 ≤ FBG < 7.0 mmol/L or 2 h PG < 11.1 mmol/L. And prediabetes was divided into 3 groups as follows: impaired fasting glucose (IFG): 6.1 ≤ FBG < 7.0 mmol/L and 2 h PG < 7.8 mmol/L; impaired glucose tolerance (IGT): FBG < 6.1 mmol/L and 7.8 ≤ 2 h PG < 11.1 mmol/L; and IFG+IGT: 6.1 ≤ FBG < 7.0 mmol/L and 7.8 ≤ 2 h PG < 11.1 mmol/L.

Based on the Chronic Kidney Disease Epidemiology Collaboration (CKD-EPI) showed as Table 1, the eGFRs were calculated [13].

According to the minimum recommendations provided by the American College of Sports Medicine and American Heart Association, we classified the participants into 3 groups based on MET hours per week as follows: <7.5 MET hours per week that equals <150 minutes per week of moderate-intensity physical activity, ≥7.5 MET hours per week <21, and ≥21 MET hours per week that equals ≥420 minutes per week of moderate-intensity physical activity on the basis of the Institute of Medicine recommendation [14, 15].

The primary outcome was the loss of renal function, which was defined as eGFR ≤ 90 mL/min/1.73^2^. The secondary outcome was the decreased eGFR as a continuous variable.

### 2.4. Statistical Analysis

All statistical analyses were performed using Empower(R) (www.empowerstats.com, X&Y Solutions Inc., Boston, MA) and R (http://www.R-project.org). A *p* value < 0.05 (2-sided) was considered statistically significant.

Data are expressed as median (25th percentile-75th percentile) for continuous variables of nonnormal distribution. Category variables are expressed as percentage (%). The continuous variables of nonnormal distribution were analyzed using the Kruskal-Wallis test. The category variables were tested using the chi-square test. Linear regression analysis was conducted to detect the relationship between different MET hours per week and eGFR as a continuous variable. A logistic regression model was built to investigate the correlation between different MET hours per week and renal dysfunction which was defined as eGFR ≤ 90 mL/min/1.73^2^, and the conventional risk variables associated with renal dysfunction loss were selected for adjustment [16, 17]. We also adjusted for confounding factors that, when added to the model, changed the matched odds ratio (OR) by at least 10%. Logistic regression model I was adjusted for age and BMI. To further correct the effects of the confounding factors, logistic regression model II was adjusted for age, sex, BMI, SBP, DBP, FBG, PBG, HbA1c, HDL, LDL, TG, GGT, pulse, region, education level, smoking status, drinking status, and medication history and family history of diabetes and tumors. According to age, sex, and different diabetic states, stratification analyses were conducted to investigate the relationships between MET hours per week and the decreased eGFR. Additional analyses and propensity score matching were performed, and the combination of the propensity score and multivariable model was used to detect an association between MET and eGFR.

## 3. Results

### 3.1. Demographic Data and Hematologic Parameters

A total of 43767 participants were included in our study. The average age was 56.84 (51.16-63.00) years. A total of 30.84% of the participants were male and 69.16% were female. DM, hypertension, and cardiovascular events were observed in 14.66%, 31.54%, and of 4.21% of the participants, respectively. A total of 8945 (20.44%) individuals had the decreased eGFR defined as an eGFR ≤ 90 mL/min/1.73^2^. We found that age, sex, region, education level, SBP, pulse, HDL, LDL, TG, FBG, PBG, eGFR, smoking status, drinking status, hypertension history, DM history, cardiovascular events, medication history, family history of tumors, and family history of DM were significantly different between different MET hours per week groups. Participant demographics and hematology data are shown in Table 2.

### 3.2. Different MET Hours per Week and the Risk of Decreased eGFR

As shown in Table 3, higher MET hours per week was associated with the risk of the decreased eGFR (7.5 to <21: OR = 1.18, 95% CI [1.09, 1.29]; ≥21: OR = 1.12, 95% CI [1.05, 1.19], *p* for trend: 0.0047). In model I, there still existed the association after further adjustment for age and BMI, and when we further adjusted for a variety of confounding factors in model II, the association between MET hours per week and the risk of the decreased eGFR remained robust (7.5 to <21: OR = 1.14, 95% CI [1.01, 1.29]; ≥21: OR = 1.24, 95% CI [1.14, 1.36], *p* for trend: <0.0001). The same trend can be detected when eGFR is a continuous variable, and this is shown in Table 3.

### 3.3. Different MET Hours per Week and the Risk of eGFR in Subgroups

**Figure 2:**
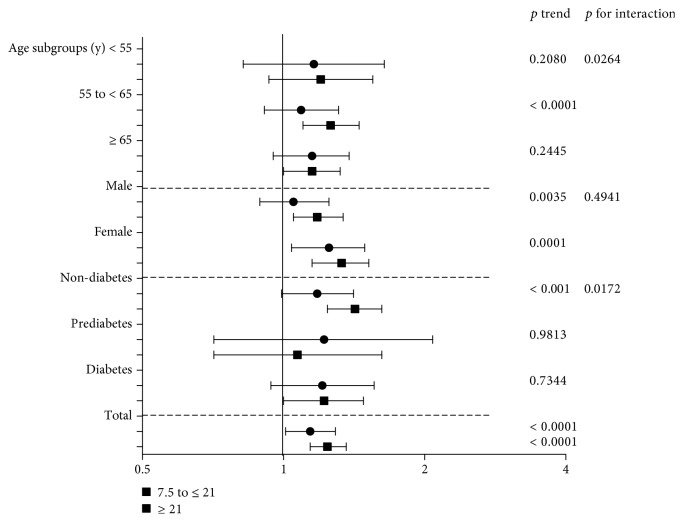
Odds ratios for logistic regression model II of the association between duration of walking quartiles and eGFR category (model II adjusted for age, gender, BMI, region, education level, SBP, DBP, pulse, LDL, HDL, TG, FBG, PBG, HbA1c, smoking status, drinking status, history of hypertension, cardiovascular disease history, diabetes history, medication history, diabetes family history, and hypertension family history).

As shown in Table 4 and Figure 2, there was a clear association between different MET hours per week and the risk of decreased eGFR in different subgroups. Compared to men expending less than 7.5 MET hours per week, those expending 7.5 to <21 MET hours per week had an OR of 1.05 (95% CI [0.89, 1.25]) and those expending ≥21 MET hours per week had a higher OR of 1.18 (95% CI [1.05, 1.34]) in model II (*p* for trend = 0.0035). Similarly, there existed a stronger association in women, and compared to men, in fully adjusted model II, the negative association between MET hours per week and continuous eGFR was significant in women (7.5 to <21: *Β* = −0.53, 95% CI [-1.00, -0.07]; ≥21: *Β* = −0.75, 95% CI [-1.09, -0.41], *p* for trend: <0.0001).

As shown in Table 4 and Figure 2, MET hours per week was significantly associated with the risk of decreased eGFR in participants aged from 55 to less than 65 years, but not in participants younger than 55 or older than 65 years. After further adjustment for confounding factors, the multivariable adjusted OR for MET hours per week from 7.5 to less than 21 and more than 21 was 1.09 (95% CI [0.91, 1.31]) and 1.26 (95% CI [1.10, 1.45]), respectively (*p* for trend < 0.0001), in participants aged from 55 to less than 65 years.

Table 4 and Figure 2 show that there was a positive relationship of MET hours per week with the risk of decreased eGFR among participants without diabetes and prediabetes, but not among participants with diabetes or prediabetes. After further adjustment for the multiple variables, the relationship was strengthened and remained significant among participants without diabetes and prediabetes (7.5 to <21: OR = 1.18, 95% CI [0.99, 1.41]; ≥21: OR = 1.42, 95% CI [1.24, 1.62], *p* for trend: <0.0001), but there was still no significant association among participants with diabetes or prediabetes. Additionally, we further investigated the association between MET hours per week and the risk of decreased eGFR among participants with prediabetes. Prediabetes was divided into 3 groups based on WHO criteria IFG, IGT, and IFG+IGT, and the results are shown in Table 5. Only among participants with IGT was there a negative association between MET hours per week and continuous eGFR (7.5 to <21: *Β* = −0.76, 95% CI [-1.78, 0.27]; ≥21: *Β* = −0.87, 95% CI [-1.64, -0.11], *p* for trend = 0.0344), but we did not investigate a relationship among participants with IFG or IFG+IGT.

The combination of the propensity score and multivariable model was used to analyze the association between MET and eGFR, including total population and subgroups. In order to further adjust for confounding factors, a logistic regression model adjusted for the propensity score was also used and the results are consistent with our previous results, which are presented in Supplementary Materials (available here).

The above results showed that the association between MET hours per week and the risk of decreased eGFR can be modified by age and diabetes status, and we found significant interactions with age and diabetes states as shown in Figure 2.

## 4. Discussion

This cross-sectional study shows that higher MET hours per week is significantly associated with the risk of decreased eGFR. After further investigation, the study indicates that the results vary in different subgroups of age and diabetic states. Compared to participants engaged in less than 7.5 MET hours per week (equivalent to less than 150 minutes per week of moderate-intensity exercise), those taking part in more than 7.5 MET hours per day (equivalent to more than 150 to less than 420 minutes or more than 420 minutes per week of moderate-intensity exercise) are more likely to have a decreased eGFR, suggesting renal dysfunction probably, especially in participants aged from 55 to less than 65 years or those without diabetes and prediabetes.

Some studies showed that physical activity is beneficial to reducing mortality [2]. However, other studies indicated that vigorous exercise has been found to be associated with an increased risk of CVD [18, 19]. Therefore, the association between different intensities of exercise and the risk of decreased eGFR is still not consistent. To the best of our knowledge, this is the first study to show that more than 7.5 MET hours per week of moderate-intensity exercise (equivalent to 150 minutes per week or more than 60 minutes per day) is associated with the risk of decreased eGFR, especially in different subgroups of age and diabetic states.

The relationship might be partially explained by the following mechanisms. Due to excessive physical activity causing oxidative stress [20, 21], there is an imbalance between the reactive oxygen species (ROS) and antioxidants produced by excessive physical activity [20, 22]. In 1978, Dillard et al. showed that physical activity led to an increase in lipid peroxidation [23]. Moreover, it could lead to an increased release of catecholamine; this, in turn, promotes the production of free radicals. In addition, a high level of physical activity is associated with temporary hypoxia in many organs, including the kidney [20]. During the course of physical activity, the blood of the visceral organs is diverted, to increase the blood supply to the active skeletal muscle and skin. After physical activity, reoxygenation occurs in hypoxic tissues. Reoxygenation and the production of ROS are related [24], and an increase in these can result in increased levels of oxidative stress and oxygen free radicals. The increased oxygen free radicals can damage lipid, protein, and DNA. ROS causes lipid peroxidation, resulting in cell membrane fluid loss and cell lysis. Also, it causes the loss of protein activity and attacks the nucleic acids which are related with DNA damage [25]. An imbalance in the production and scavenging of ROS can lead to cell dysfunction.

In some studies, excessive physical activity was associated with the low degree of inflammation. The elevated levels of neutrophils, monocytes, and leucocytes indicated a slightly greater degree of inflammation after high-intensity physical activity [20]. Long duration of physical activity may lead to greater metabolic demands and the increased release of stress hormones (catecholamines, growth hormones, and cortisol). The increased levels of stress hormones may affect the activation and mobilization of immune cells [26, 27]. Sahl et al. showed that among elderly adults, excessive physical activity increased plasma IL-6 concentrations and low-grade inflammation [28]. However, some studies found that physical activity reduced inflammation [29, 30]. An alternative explanation is that, during excessive physical activity, oxidative stress surpasses the antioxidant defense, resulting in a weakened anti-inflammatory effect, although, in the case of low-intensity physical activity, the antioxidant stress system defense may meet an increased production of ROS. Oxidative stress induces podocyte apoptosis, resulting in glomerular sclerosis and the activation of signal transduction, involved in renal tubular extracellular matrix secretion, to promote interstitial fibrosis [31]. Inflammation plays a key role in the loss of renal function.

In addition, renal tubular hypoxia is one of the important reasons of renal dysfunction. If physical activity is excessive, such as more than 60 minutes per day of moderate-intensity exercise, visceral hypoperfusion may occur. Kidney hypoperfusion is likely due to renal tubular hypoxia, and more than 21 MET hours per week might cause a decrease in renal blood flow leading to low perfusion of the kidneys, resulting in a decreased eGFR [32].

The above results of this cross-sectional study highlight 2 significant points for renal function protection. Firstly, long duration of exercise (more than 150 minutes per week of moderate-intensity or more than 60 minutes per day of moderate-intensity exercise) might not be appropriate for everyone. Individuals' exercise plan should be considered based on individuals' health conditions. Secondly, people aged from 55 to less than 65 years or without diabetes and prediabetes should maintain less than 150 minutes per week of moderate-intensity exercise to keep healthy. However, for those aged younger than 55 and older than 65 years or with prediabetes and diabetes, 60 minutes per day of moderate-intensity exercise may be a good recommendation to help them keep fit.

### 4.1. Perspective and Shortcomings

Our study is unique as the sample size was large. We conducted stratified analyses that fully explored not only the relationship between different MET hours per week and decreased eGFR but also the interaction. Though the confounding factors were adequately adjusted for as possible as we can, particularly in terms of sociological factors, region, and education level, it is possible that unmeasured variables are involved in that association between MET hours per week and the risk of decreased eGFR. Due to the study design, the changes occurring while carrying out the exercise were not monitored and the mechanism underlying the association between high MET hours per week and decreased eGFR was not explored.

## 5. Conclusion

In conclusion, more than 7.5 MET hours per week (equivalent to more than 150 minutes per week of moderate-intensity exercise) is associated with decreased eGFR. There is a clear association in participants aged ≥55 and <65 years and populations without diabetes and prediabetes, but not in populations aged <55 years or ≥65 years and those with prediabetes or diabetes. Duration of exercise may need to be individualized, to ensure optimal treatment in subgroups of different diabetic states. The positive effect of exercise may depend on the optimal duration of exercise based on individuals' conditions. Should we just tell our patients to do some exercise to help them keep fit or provide individuals' exercise plan in detail based on their condition?

## Figures and Tables

**Table 1:** 

Sex	Age (years)	Formula (mL/min/1.73^2^)
Female	≤62	eGFR = 144 × (serum creatinine/0.7)^−0.329^ × (0.993)^age^
	>62	eGFR = 144 × (serum creatinine/0.7)^−1.209^ × (0.993)^age^
Male	≤62	eGFR = 141 × (serum creatinine/0.7)^−0.411^ × (0.993)^age^
	>62	eGFR = 141 × (serum creatinine/0.7)^−1.209^ × (0.993)^age^

**Table 2:** Characteristics of the participants in different groups of MET hours per week.

Variables	Total	Physical activity (MET hours per week)			
		<7.5	7.5 ≥ MET < 21	≥21	*p* value
*N*	43767	8258	5874	29635	
Age (y)	56.84 (51.16-63.00)	56.61 (50.52-62.97)	57.49 (51.92-64.09)	56.80 (51.12-62.83)	<0.001
Male (%)	13497 (30.84%)	2392 (28.97%)	1697 (28.89%)	9408 (31.75%)	<0.001
BMI (kg/m^2^)	24.13 (21.98-26.42)	24.05 (21.83-26.49)	24.16 (22.04-26.46)	24.13 (22.02-26.39)	0.881
SBP (mmHg)	128.00 (116.00-143.00)	128.00 (116.00-143.00)	129.00 (116.00-144.00)	128.00 (115.00-142.00)	<0.001
DBP (mmHg)	76.00 (70.00-84.00)	76.00 (70.00-84.00)	77.00 (70.00-84.00)	76.00 (70.00-84.00)	0.237
Pulse (bpm)	78.00 (71.00-86.00)	78.00 (72.00-86.00)	78.00 (71.00-86.00)	78.00 (71.00-86.00)	<0.001
HDL (mmol/L)	1.29 (1.08-1.53)	1.30 (1.08-1.55)	1.29 (1.08-1.51)	1.29 (1.08-1.53)	<0.001
LDL (mmol/L)	2.90 (2.31-3.53)	2.90 (2.29-3.52)	2.90 (2.29-3.54)	2.95 (2.37-3.56)	<0.001
TG (mmol/L)	1.30 (0.93-1.88)	1.30 (0.93-1.88)	1.35 (0.95-1.93)	1.30 (0.93-1.87)	0.048
GGT (mmol/L)	20.00 (14.00-30.00)	20.00 (14.00-31.00)	20.00 (14.00-31.00)	20.00 (14.00-30.00)	0.131
FBG (mmol/L)	5.46 (5.09-5.93)	5.46 (5.10-5.92)	5.45 (5.08-5.92)	5.46 (5.09-5.94)	0.005
PBG (mmol/L)	7.08 (5.86-8.81)	7.09 (5.91-8.86)	7.10 (5.83-8.80)	7.07 (5.85-8.80)	0.015
HbA1c (%)	5.80 (5.50-6.10)	5.80 (5.50-6.10)	5.80 (5.50-6.10)	5.80 (5.50-6.10)	0.614
eGFR (mL/min/1.73^2^)	100.10 (92.43-106.43)	101.00 (93.56-107.24)	99.81 (91.92-105.90)	99.90 (92.25-106.27)	<0.001
>90	34822 (79.56%)	6699 (81.12%)	4605 (78.40%)	23518 (79.36%)	
≤90	8945 (20.44%)	1559 (18.88%)	1269 (21.60%)	6117 (20.64%)	
Region (%)					<0.001
Northern	19484 (44.52%)	3227 (39.08%)	2721 (46.32%)	13536 (45.68%)	
Southern	24283 (55.48%)	5031 (60.92%)	3153 (53.68%)	16099 (54.32%)	
Education (%)					<0.001
Illiteracy	1911 (4.37%)	557 (6.74%)	204 (3.47%)	1150 (3.88%)	
Primary school	5187 (11.85%)	1150 (3.88%)	1150 (3.88%)	1150 (3.88%)	
Junior high school	14985 (34.24%)	2876 (34.83%)	1803 (30.69%)	10306 (34.78%)	
Senior high school	15780 (36.05%)	2643 (32.01%)	2176 (37.04%)	10961 (36.99%)	
College	5904 (13.49%)	1053 (12.75%)	1105 (18.81%)	3746 (12.64%)	
Smoking status (%)					<0.001
No	37641 (86.00%)	7156 (86.66%)	5110 (86.99%)	25375 (85.63%)	
Occasional smokers	1047 (2.39%)	156 (1.89%)	122 (2.08%)	769 (2.59%)	
Regular smokers	5079 (11.60%)	946 (11.46%)	642 (10.93%)	3491 (11.78%)	
Drinking status (%)					<0.001
No	32375 (73.97%)	6280 (76.05%)	4374 (74.46%)	21721 (73.30%)	
Occasional drinkers	8348 (19.07%)	1428 (17.29%)	1092 (18.59%)	5828 (19.67%)	
Regular drinkers	3044 (6.96%)	550 (6.66%)	408 (6.95%)	2086 (7.04%)	
Prevalence of diseases (%)					
Diabetes mellitus	6418 (14.66%)	1294 (15.67%)	798 (13.59%)	4326 (14.60%)	<0.001
Hypertension	13804 (31.54%)	2614 (31.65%)	1990 (33.88%)	9200 (31.04%)	<0.001
Cardiovascular events	1842 (4.21%)	312 (3.78%)	296 (5.04%)	1234 (4.16%)	<0.001
Medication (%)					
Antihypertensive drugs	5955 (13.61%)	905 (10.96%)	990 (16.85%)	4060 (13.70%)	<0.001
Hypoglycemic drugs	55 (0.13%)	17 (0.21%)	2 (0.03%)	36 (0.12%)	<0.001
Lipid-lowering drugs	399 (0.91%)	64 (0.78%)	74 (1.26%)	261 (0.88%)	0.007
Family history of disease (%)					
Family history of diabetes	6593 (15.06%)	1018 (12.33%)	1040 (17.71%)	4535 (15.30%)	<0.001
Family history of tumor	6109 (13.96%)	994 (12.04%)	975 (16.60%)	4140 (13.97%)	<0.001

Data of characteristics expressed as median (25^th^ percentile-75^th^ percentile) for continuous variables of nonnormal distribution and percentage (%) for categorical variables. BMI: body mass index; SBP: systolic blood pressure; DBP: diastolic blood pressure; eGFR: estimated glomerular filtration rate; TG: triglyceride; LDL: low-density lipoprotein cholesterol; HDL: high-density lipoprotein cholesterol; GGT: gamma-glutamyltranspeptidase; FBG: 0-hour fasting blood glucose; PBG: 2-hour postprandial blood glucose. An expenditure of 7.5 MET hours per week is equivalent to 150 minutes per week of moderate-intensity physical activity, the minimum recommended by the federal government; 21 MET hours per week is equivalent to 60 minutes per day (420 min/week) of moderate-intensity physical activity, recommended by the Institute of Medicine.

**Table 3:** Correlation analysis between physical activity (MET hours per week) and the risk of decreased eGFR.

Variable	Nonadjusted	*p* trend	Model I	*p* trend	Model II	*p* trend
eGFR category						
Physical activity (MET hours per week)		0.0047		<0.0001		<0.0001
<7.5	1.0		1.0		1.0	
7.5 to <21	1.18 (1.09, 1.29)		1.12 (1.01, 1.23)		1.14 (1.01, 1.29)	
≥21	1.12 (1.05, 1.19)		1.27 (1.18, 1.37)		1.24 (1.14, 1.36)	
Continuous eGFR (mL/min/1.73 m^2^)						
Physical activity (MET hours per week)		0.0009		<0.0001		<0.0001
<7.5	0		0		0	
7.5 to <21	-1.38 (-1.90, -0.86)		-0.58 (-1.04, -0.12)		-0.45 (-0.85, -0.04)	
≥21	-0.82 (-1.20, -0.44)		-0.93 (-1.26, -0.60)		-0.64 (-0.94, -0.34)	

Nonadjusted model. Model I adjusted for age and BMI; model II adjusted for age, sex, BMI, region, education level, SBP, DBP, pulse, LDL, HDL, TG, FBG, PBG, HbA1c, GGT, smoking status, drinking status, history of hypertension, cardiovascular history, diabetes history, medication history, and family history of diabetes and tumors.

**Table 4:** Correlation analysis between physical activity (MET hours per week) and the risk of decreased eGFR in subgroups.

	Variable	Nonadjusted	*p* trend	Model I	*p* trend	Model II	*p* trend
Male	eGFR category						
	Physical activity (MET hours per week)		0.2692		0.0310		0.0035
	<7.5	1.0		1.0		1.0	
	7.5 to <21	1.27 (1.12, 1.44)		1.05 (0.90, 1.23)		1.05 (0.89, 1.25)	
	≥21	1.10 (1.00, 1.20)		1.13 (1.01, 1.26)		1.18 (1.05, 1.34)	
	Continuous eGFR (mL/min/1.73 m^2^)						
	Physical activity (MET hours per week)		0.1464		0.8981		0.1057
	<7.5	0		0		0	
	7.5 to <21	-1.95 (-2.95, -0.96)		-0.18 (-0.94, 0.59)		-0.16 (-0.92, 0.60)	
	≥21	0.08 (-0.63, 0.80)		-0.00 (-0.56, 0.55)		-0.43 (-0.98, 0.13)	
Female	eGFR category						
	Physical activity (MET hours per week)		0.5679		<0.0001		0.0001
	<7.5	1.0		1.0		1.0	
	7.5 to <21	1.19 (1.04, 1.37)		1.25 (1.05, 1.48)		1.25 (1.04, 1.49)	
	≥21	1.01 (0.91, 1.12)		1.34 (1.17, 1.52)		1.33 (1.15, 1.52)	
	Continuous eGFR (mL/min/1.73 m^2^)						
	Physical activity (MET hours per week)		0.0143		0.0001		<0.0001
	<7.5	0		0		0	
	7.5 to <21	-1.16 (-1.68, -0.64)		-0.74 (-1.21, -0.26)		-0.53 (-1.00, -0.07)	
	≥21	-0.63 (-1.01, -0.25)		-0.74 (-1.08, -0.39)		-0.75 (-1.09, -0.41)	
age < 55 years	eGFR category						
	Physical activity (MET hours per week)		0.0351		0.0549		0.2080
	<7.5	1.0		1.0		1.0	
	7.5 to <21	1.19 (0.89, 1.60)		1.11 (0.82, 1.50)		1.16 (0.82, 1.64)	
	≥21	1.26 (1.02, 1.57)		1.23 (0.99, 1.53)		1.20 (0.93, 1.55)	
	Continuous eGFR (mL/min/1.73 m^2^)						
	Physical activity (MET hours per week)		0.0172		0.1714		0.0031
	<7.5	0		0		0	
	7.5 to <21	-0.94 (-1.39, -0.49)		-0.37 (-0.77, 0.03)		-0.53 (-0.84, -0.22)	
	≥21	-0.50 (-0.82, -0.19)		-0.24 (-0.52, 0.04)		-0.30 (-0.52, -0.08)	
Age 55-64 years	eGFR category						
	Physical activity (MET hours per week)		<0.0001		<0.0001		<0.0001
	<7.5	1.0		1.0		1.0	
	7.5 to <21	1.05 (0.92, 1.21)		1.04 (0.90, 1.20)		1.09 (0.91, 1.31)	
	≥21	1.22 (1.11, 1.35)		1.24 (1.12, 1.38)		1.26 (1.10, 1.45)	
	Continuous eGFR (mL/min/1.73 m^2^)						
	Physical activity (MET hours per week)		<0.0001		<0.0001		<0.0001
	<7.5	0		0		0	
	7.5 to <21	-0.69 (-1.35, -0.02)		-0.71 (-1.36, -0.06)		-0.70 (-1.28, -0.12)	
	≥21	-1.22 (-1.71, -0.73)		-1.22 (-1.71, -0.74)		-1.06 (-1.49, -0.63)	
age ≥ 65 years	eGFR category						
	Physical activity (MET hours per week)		0.7888		0.2445		0.7046
	<7.5	1.0		1.0		1.0	
	7.5 to <21	1.11 (0.96, 1.28)		1.18 (1.01, 1.37)		1.15 (0.95, 1.38)	
	≥21	1.05 (0.94, 1.17)		1.23 (1.10, 1.38)		1.15 (1.00, 1.32)	
	Continuous eGFR (mL/min/1.73 m^2^)						
	Physical activity (MET hours per week)		0.6376		0.7733		0.9339
	<7.5	0		0		0	
	7.5 to <21	-0.52 (-2.24, 1.20)		-1.15 (-2.80, 0.50)		-0.31 (-1.73, 1.11)	
	≥21	-0.51 (-1.81, 0.78)		-2.43 (-3.69, -1.17)		-1.22 (-2.32, -0.13)	
Nondiabetes	eGFR category						
	Physical activity (MET hours per week)		0.0001		<0.0001		<0.0001
	<7.5	1.0		1.0		1.0	
	7.5 to <21	1.23 (1.09, 1.39)		1.13 (0.98, 1.30)		1.18 (0.99, 1.41)	
	≥21	1.21 (1.11, 1.32)		1.39 (1.25, 1.55)		1.42 (1.24, 1.62)	
	Continuous eGFR (mL/min/1.73 m^2^)						
	Physical activity (MET hours per week)		0.0001		<0.0001		<0.0001
	<7.5	0		0		0	
	7.5 to <21	-1.48 (-2.11, -0.85)		-0.51 (-1.04, 0.02)		-0.38 (-0.85, 0.09)	
	≥21	-1.06 (-1.52, -0.60)		-1.07 (-1.46, -0.68)		-0.78 (-1.12, -0.44)	
Prediabetes	eGFR category						
	Physical activity (MET hours per week)		0.8518		0.0113		0.9813
	<7.5	1.0		1.0		1.0	
	7.5 to <21	1.21 (1.05, 1.39)		1.17 (0.99, 1.38)		1.22 (0.71, 2.08)	
	≥21	1.05 (0.94, 1.17)		1.19 (1.05, 1.35)		1.07 (0.71, 1.62)	
	Continuous eGFR (mL/min/1.73 m^2^)						
	Physical activity (MET hours per week)		0.0720		0.0025		0.2905
	<7.5	0		0		0	
	7.5 to <21	-1.69 (-2.70, -0.68)		-0.97 (-1.89, -0.05)		-1.70 (-3.46, 0.06)	
	≥21	-0.94 (-1.69, -0.19)		-1.11 (-1.79, -0.43)		-0.80 (-2.12, 0.51)	
Diabetes	eGFR category						
	Physical activity (MET hours per week)		0.7555		0.1762		0.7344
	<7.5	1.0		1.0		1.0	
	7.5 to <21	1.05 (0.86, 1.30)		1.03 (0.92, 1.16)		1.21 (0.94, 1.56)	
	≥21	1.03 (0.89, 1.19)		1.07 (0.96, 1.19)		1.22 (1.00, 1.48)	
	Continuous eGFR (mL/min/1.73 m^2^)						
	Physical activity (MET hours per week)		0.5247		0.8804		0.4650
	<7.5	0		0		0	
	7.5 to <21	-0.53 (-2.21, 1.15)		-0.14 (-1.69, 1.41)		0.12 (-1.26, 1.50)	
	≥21	0.26 (-0.93, 1.44)		0.05 (-1.05, 1.15)		0.33 (-0.65, 1.31)	

Nonadjusted model for none. Model I adjusted for age and BMI. Model II adjusted for age, sex, BMI, region, education level, SBP, DBP, pulse, LDL, HDL, TG, FBG, PBG, HbA1c, GGT, smoking status, drinking status, history of hypertension, cardiovascular history, diabetes history, medication history, and family history of diabetes and tumor.

**Table 5:** Correlation analysis between physical activity (MET hours per week) and the risk of decreased eGFR in IFG, IGT, and IFG+IGT groups.

	Variable	Nonadjusted	*p* trend	Model I	*p* trend	Model II	*p* trend
IFG	eGFR category						
	Physical activity (MET hours per week)		0.5640		0.6828		0.5091
	<7.5	1.0		1.0		1.0	
	7.5 to <21	1.14 (0.80, 1.63)		0.88 (0.58, 1.34)		1.22 (0.71, 2.08)	
	≥21	0.96 (0.74, 1.25)		0.90 (0.66, 1.23)		1.07 (0.71, 1.62)	
	Continuous eGFR (mL/min/1.73 m^2^)						
	Physical activity (MET hours per week)		0.9681		0.7192		0.4421
	<7.5	0		0		0	
	7.5 to <21	-2.11 (-4.37, 0.16)		-0.60 (-2.61, 1.41)		-1.70 (-3.46, 0.06)	
	≥21	-0.38 (-2.05, 1.29)		-0.23 (-1.71, 1.25)		-0.80 (-2.12, 0.51)	
IGT	eGFR category						
	Physical activity (MET hours per week)		0.5841		0.0030		0.0685
	<7.5	1.0		1.0		1.0	
	7.5 to <21	1.19 (1.00, 1.43)		1.21 (0.98, 1.49)		1.21 (0.94, 1.56)	
	≥21	1.07 (0.94, 1.22)		1.28 (1.09, 1.49)		1.22 (1.00, 1.48)	
	Continuous eGFR (mL/min/1.73 m^2^)						
	Physical activity (MET hours per week)		0.0575		0.0031		0.0344
	<7.5	0		0		0	
	7.5 to <21	-1.55 (-2.83, -0.27)		-0.99 (-2.15, 0.17)		-0.76 (-1.78, 0.27)	
	≥21	-1.11 (-2.05, -0.16)		-1.38 (-2.24, -0.52)		-0.87 (-1.64, -0.11)	
IFG+IGT	eGFR category						
	Physical activity (MET hours per week)		0.4556		0.6495		0.9494
	<7.5	1.0		1.0		1.0	
	7.5 to <21	1.30 (0.93, 1.81)		1.21 (0.82, 1.78)		1.37 (0.85, 2.21)	
	≥21	1.04 (0.80, 1.34)		1.09 (0.81, 1.46)		1.06 (0.74, 1.53)	
	Continuous eGFR (mL/min/1.73 m^2^)						
	Physical activity (MET hours per week)		0.7212		0.3478		0.3827
	<7.5	0		0		0	
	7.5 to <21	-1.79 (-4.18, 0.60)		-1.10 (-3.27, 1.06)		-0.78 (-2.69, 1.14)	
	≥21	-0.66 (-2.44, 1.11)		-0.87 (-2.48, 0.74)		-0.72 (-2.15, 0.71)	

## Data Availability

The datasets used to support this study are not freely available considering participants' privacy protection.

## Supplementary Materials

Supplementary Materials Table S1: characteristics of the participants in a matched cohort. Table S2: correlation analysis between physical activity (MET hours per week) and the risk of decreased eGFR in covariate adjustment using the propensity score (CAPS) model. Table S3: correlation analysis between physical activity (MET hours per week) and the risk of decreased eGFR in subgroups using the CAPS model. Table S4: correlation analysis between physical activity (MET hours per week) and the risk of decreased eGFR in IFG, IGT, and IFG+IGT groups using the CAPS model.
