# Supplementary material for: Association between Duration of Exercise (MET Hours per Week) and the Risk of Decreased eGFR: A Cross-Sectional Study Based on a Large Chinese Population
Source: J Diabetes Res. 2019 Mar 21;2019:5874603. doi: 10.1155/2019/5874603 (PMC6451799; doi:10.1155/2019/5874603)
Supplement: Supplementary Materials — Table S1: characteristics of the participants in a matched cohort. Table S2: correlation analysis between physical activity (MET hours per week) and the risk of decreased eGFR in covariate adjustment using the propensity score (CAPS) model. Table S3: correlation analysis between physical activity (MET hours per week) and the risk of decreased eGFR in subgroups using the CAPS model. Table S4: correlation analysis between physical activity (MET hours per week) and the risk of decreased eGFR in IFG, IGT, and IFG+IGT groups using the CAPS model. [file 5874603.f1.pdf]

Table S1. Characteristics of the participants in matched cohort

Variables	Total	Physical Activity (MET hours per week)		P-value
		<7.5	≥7.5	
N	23825	4765	19060	
Age (y)	56.10 (50.60- 61.93)	55.8 (50.0-61.7)	56.2 (50.7-62.0)	0.009
Male (%)	7480 (31.40%)	1441 (30.2%)	6039 (31.7%)	0.009
BMI (kg/m2)	24.35 (22.25- 26.70)	24.3 (22.1-26.7)	24.4 (22.3-26.7)	0.227
SBP (mmHg)	129.00 (117.00-144.00)	129.0 (117.0-143.0)	129.0 (117.0-144.0)	0.301
DBP (mmHg)	77.00 (70.00- 85.00)	77.0 (70.0-84.0)	77.0 (70.0-85.0)	0.113
Pulse (bpm)	78.00 (71.00- 86.00)	78.0 (72.0-86.0)	78.0 (71.0-86.0)	0.168
HDL (mmol/L)	1.30 (1.09- 1.54)	1.3 (1.1-1.5)	1.3 (1.1-1.5)	0.865
LDL (mmol/L)	2.96 (2.36- 3.59)	3.0 (2.4-3.6)	3.0 (2.4-3.6)	0.914
TG (mmol/L)	1.28 (0.91- 1.84)	1.3 (0.9-1.9)	1.3 (0.9-1.8)	0.380
GGT (mmol/L)	20.00 (15.00- 31.00)	20.0 (15.0-32.0)	20.0 (15.0-31.0)	0.065
FBG (mmol/L)	5.49 (5.12- 5.96)	5.5 (5.1-6.0)	5.5 (5.1-6.0)	0.625
PBG (mmol/L)	7.03 (5.87- 8.71)	7.0 (5.9-8.7)	7.0 (5.9-8.7)	0.657
HbA1c (%)	5.80 (5.50- 6.10)	5.8 (5.5-6.1)	5.8 (5.5-6.1)	0.518
eGFR (ml/min/1.73²)				0.004
> 90	19386 (81.37%)	3946 (82.8%)	15440 (81.0%)	
≤ 90	4439 (18.63%)	819 (17.2%)	3620 (19.0%)	
Region (%)				1.000
Northern	10965 (46.02%)	2193 (46.0%)	8772 (46.0%)	
Southern	12860 (53.98%)	2572 (54.0%)	10288 (54.0%)	
Education (%)				0.067
illiteracy	656 (2.75%)	153 (3.2%)	503 (2.6%)	
primary school	2356 (9.89%)	477 (10.0%)	1879 (9.9%)	

**junior high school**	8459 (35.50%)	1649 (34.6%)	6810 (35.7%)	
**senior high school**	9021 (37.86%)	1783 (37.4%)	7238 (38.0%)	
**college**	3333 (13.99%)	703 (14.8%)	2630 (13.8%)	
**Smoking status (%)**				0.218
**no**	20360 (85.46%)	4088 (85.8%)	16272 (85.4%)	
**occasional smokers**	594 (2.49%)	102 (2.1%)	492 (2.6%)	
**regular smokers**	2871 (12.05%)	575 (12.1%)	2296 (12.0%)	
**Drinking status (%)**				0.373
**no**	17294 (72.59%)	3446 (72.3%)	13848 (72.7%)	
**occasional drinkers**	4743 (19.91%)	977 (20.5%)	3766 (19.8%)	
**regular drinkers**	1788 (7.50%)	342 (7.2%)	1446 (7.6%)	
**Prevalence of diseases (%)**				
**Diabetes mellitus**	3119 (13.09%)	628 (13.2%)	2491 (13.1%)	0.840
**Hypertension**	8165 (34.27%)	1596 (33.5%)	6569 (34.5%)	0.207
**Cardiovascular events**	969 (4.07%)	187 (3.92%)	782 (4.10%)	0.577
**Family history of disease (%)**				
**Family history of diabetes**	4009 (16.83%)	753 (15.8%)	3256 (17.1%)	0.035
**Family history of tumor**	3613 (15.16%)	699 (14.7%)	2914 (15.3%)	0.287

Table S2. Correlation analysis between physical activity (MET hours per week) and the risk of decreased eGFR in covariate adjustment using propensity score (CAPS) model

Variable	Non-adjusted	P-value	Model I	P-value
**eGFR Category**				
**Physical Activity (MET hours per week)**				
**<7.5**	1.0		1.0	
**≥7.5**	1.1 (1.0, 1.2)	0.0038	1.10 (1.01, 1.19)	0.0352
**Continuous eGFR (ml/min/1.73 m^2)**				
**Physical Activity (MET hours per week)**				
**<7.5**	0		0	
**≥7.5**	-0.61 (-1.07, -0.16)	0.0084	-0.42 (-0.88, 0.04)	0.0704

Non-adjusted model

Model I adjusted for： propensity score

Table S3. Correlation analysis between physical activity (MET hours per week) and the risk of decreased eGFR in subgroups using CAPS model

	Variable	Non-adjusted	P-value	Model I	P-value
**Age <55 years**	eGFR Category				
	Physical Activity (MET hours per week)				
	<7.5	1.0		1.0	
	≥7.5	1.33 (0.99, 1.79)	0.0552	1.30 (0.97, 1.75)	0.0832
	Continuous eGFR (ml/min/1.73 m^2)				

	Physical Activity (MET hours per week)				
	<7.5	0		0	
	⩾7.5	-0.25 (-0.61, 0.11)	0.1769	-0.17 (-0.53, 0.19)	0.3500
**Age 55-64 years**	eGFR Category				
	Physical Activity (MET hours per week)				
	<7.5	1.0		1.0	
	⩾7.5	1.20 (1.04, 1.38)	0.0118	1.16 (1.00, 1.33)	0.0459
	Continuous eGFR (ml/min/1.73 m^2)				
	Physical Activity (MET hours per week)				
	<7.5	0		0	
	⩾7.5	-0.85 (-1.44, -0.27)	0.0042	-0.69 (-1.27, -0.10)	0.0223
**Age ≥65 years**	eGFR Category				
	Physical Activity (MET hours per week)				
	<7.5	1.0		1.0	
	⩾7.5	1.05 (0.87, 1.28)	0.5991	1.03 (0.85, 1.25)	0.7449
	Continuous eGFR (ml/min/1.73 m^2)				
	Physical Activity (MET hours per week)				
	<7.5	0		0	
	⩾7.5	0.02 (-1.79, 1.83)	0.9839	0.13 (-1.67, 1.93)	0.8868
**Non-diabetes**	eGFR Category				
	Physical Activity (MET hours per week)				
	<7.5	1.0		1.0	
	⩾7.5	1.31 (1.15, 1.49)	<0.0001	1.25 (1.10, 1.43)	0.0007
	Continuous eGFR (ml/min/1.73 m^2)				
	Physical Activity (MET hours per week)				
	<7.5	0		0	

	⩾7.5	-1.11 (-1.65, -0.58)	<0.0001	-0.91 (-1.44, -0.38)	0.0008
**Prediabetes**	eGFR Category				
	Physical Activity (MET hours per week)				
	<7.5	1.0		1.0	
	⩾7.5	1.02 (0.88, 1.20)	0.7684	1.00 (0.85, 1.17)	0.9706
	Continuous eGFR (ml/min/1.73 m^2)				
	Physical Activity (MET hours per week)				
	<7.5	0		0	
	⩾7.5	-0.37 (-1.28, 0.54)	0.4259	-0.21 (-1.12, 0.70)	0.6546
**Diabetes**	eGFR Category				
	Physical Activity (MET hours per week)				
	<7.5	1.0		1.0	
	⩾7.5	0.68 (0.53, 0.88)	0.0035	0.65 (0.50, 0.85)	0.0012
	Continuous eGFR (ml/min/1.73 m^2)				
	Physical Activity (MET hours per week)				
	<7.5	0		0	
	⩾7.5	1.07 (-0.44, 2.58)	0.1635	1.25 (-0.26, 2.76)	0.1055

Non-adjusted model

Model I adjusted for： propensity score

Table S4. Correlation analysis between physical activity (MET hours per week) and the risk of decreased eGFR in IFG, IGT and IFG+IGT groups using CAPS model

	Variable	Non-adjusted	P-value	Model I	P-value
**IFG**	eGFR Category				
	Physical Activity (MET hours per week)				

	<7.5	1.0		1.0	
	≥7.5	1.41 (0.79, 2.53)	0.2506	1.36 (0.76, 2.45)	0.3017
	Continuous eGFR (ml/min/1.73 m^2)				
	Physical Activity (MET hours per week)				
	<7.5	0		0	
	≥7.5	-0.91 (-3.00, 1.19)	0.3958	-0.68 (-2.76, 1.39)	0.5194
**IGT**	eGFR Category				
	Physical Activity (MET hours per week)				
	<7.5	1.0		1.0	
	≥7.5	0.95 (0.77, 1.18)	0.6615	0.94 (0.76, 1.16)	0.5547
	Continuous eGFR (ml/min/1.73 m^2)				
	Physical Activity (MET hours per week)				
	<7.5	0		0	
	≥7.5	-0.45 (-1.59, 0.69)	0.4386	-0.33 (-1.47, 0.81)	0.5710
**IFG+IGT**	eGFR Category				
	Physical Activity (MET hours per week)				
	<7.5	1.0		1.0	
	≥7.5	0.95 (0.54, 1.65)	0.8468	0.92 (0.53, 1.62)	0.7848
	Continuous eGFR (ml/min/1.73 m^2)				
	Physical Activity (MET hours per week)				
	<7.5	0		0	
	≥7.5	0.42 (-1.91, 2.75)	0.7214	0.71 (-1.62, 3.03)	0.5512

Non-adjusted model

Model I adjusted for： propensity score
